# Physician Engagement in Addressing Health-Related Social Needs and Burnout

**DOI:** 10.1001/jamanetworkopen.2024.52152

**Published:** 2024-12-30

**Authors:** Masami Tabata-Kelly, Xiaochu Hu, Michael J. Dill, Philip M. Alberti, Karen Bullock, William Crown, Malika Fair, Peter May, Pilar Ortega, Jennifer Perloff

**Affiliations:** 1 The Heller School for Social Policy and Management, Brandeis University, Waltham, Massachusetts; 2 Center for Surgery and Public Health, Brigham and Women’s Hospital, Boston, Massachusetts; 3 Association of American Medical Colleges, Washington, DC; 4 School of Social Work, Boston College, Boston, Massachusetts; 5 Cicely Saunders Institute of Palliative Care, Policy and Rehabilitation, King’s College London, London, United Kingdom; 6 School of Medicine, Trinity College Dublin, Dublin, Ireland; 7 Accreditation Council for Graduate Medical Education, Chicago, Illinois; 8 University of Illinois College of Medicine, Chicago

## Abstract

**Importance:**

Previous research suggests that a greater capacity of health care organizations to address patients’ health-related social needs (HRSNs) is associated with lower physician burnout. However, individual physician-level engagement in addressing HRSNs has not been fully characterized, and its association with physician burnout remains understudied.

**Objective:**

To characterize physicians’ engagement in addressing HRSNs and examine its association with burnout.

**Design, Setting, and Participants:**

This cross-sectional study used the 2022 Association of American Medical Colleges National Sample Survey of Physicians (NSSP), a nationally representative survey of active, practicing physicians in the US conducted from May to November 2022 that measured a diverse array of physician workforce characteristics.

**Main Outcome and Measures:**

HRSN engagement was defined by physicians’ responses to the NSSP item, “During the past 12 months, how often did you spend work time helping your patients meet their social needs?” Engagement levels were categorized as no engagement, low to moderate engagement (monthly or <1 time per month), or high engagement (weekly or daily). Burnout was measured using a single-item measure from the emotional exhaustion domain of the Maslach Burnout Inventory (high was defined as weekly or more). HRSN engagement was examined by physician characteristics, and multivariate logistic regression was conducted to explore associations between HRSN engagement and burnout. Data were weighted by age, gender, international medical graduate status, and specialty group.

**Results:**

In the study cohort of 5447 physicians, the mean (SD) age was 50.9 (11.7) years, and 3735 (68.6%) identified as men or transgender men. Overall, 34.3% of physicians reported high HRSN engagement, with variability based on physician characteristics. Compared with no HRSN engagement, low to moderate HRSN engagement (adjusted odds ratio [AOR], 1.33; 95% CI, 1.03-1.72; *P* = .03) and high HRSN engagement (AOR, 1.72; 95% CI, 1.39-2.27; *P* < .001) were significantly associated with high burnout.

**Conclusions and Relevance:**

In this cross-sectional study of 5447 nationally representative physicians in the US, 34.3% regularly dedicated time to addressing HRSNs. The study identified variability in physicians’ engagement in addressing HRSNs and found that higher engagement was associated with a greater likelihood of burnout. The findings suggest the need for thorough assessment of the potential unintended consequences of physicians’ engagement in addressing HRSNs on their well-being.

## Key Points

**Question:**

What are the characteristics of physicians’ engagement in addressing health-related social needs (HRSNs), and is this engagement associated with burnout?

**Findings:**

In this cross-sectional study of 5447 nationally representative physicians in the US, nearly 35% regularly dedicated their time to addressing HRSNs. There was variability in physicians’ engagement levels, and higher engagement was associated with higher burnout.

**Meaning:**

These findings suggest the need for thorough assessment of the potential unintended consequences of physicians’ engagement in addressing HRSNs on their well-being.

## Teaser

This cross-sectional study examines physician characteristics associated with engagement in addressing patients’ health-related social needs and whether this engagement is associated with burnout.

## Introduction

Addressing unmet health-related social needs (HRSNs), such as housing instability, food insecurity, transportation barriers, and utility challenges, that adversely affect individuals’ health and health care is a shared responsibility across sectors and contributes to advancing health equity and population health.^1,2,3,4^ In the US health care sector, there are ongoing efforts to create a more coordinated system that integrates social care to address HRSNs and reduce health inequities. For example, Medicaid innovations through Section 1115 demonstration waivers have supported states in implementing various pilot interventions aimed at addressing HRSNs by facilitating collaborations between health care and community-based organizations, as seen in New York State’s HRSN initiatives, which establish the infrastructure for social care networks.^5,6,7,8^ In 2024, the Centers for Medicare & Medicaid Services (CMS) took steps to advance the integration of social care into the health care system through incentive programs, including updating the Physician Fee Schedule to provide compensation for physicians addressing HRSNs.^9^

For physicians, addressing HRSNs in clinical practice may entail tasks such as screening and connecting patients to social services.^10^ However, physicians’ engagement in addressing HRSNs has not been fully characterized, and there is also limited understanding of how different subgroups of physicians spend time addressing HRSNs. Previous studies have indicated a potential inverse relationship between a health care organization’s capacity (eg, organizational resources to coordinate with social services and community resources and availability of collocated clinicians, such as social workers) to address patients’ HRSNs and physician burnout.^11,12,13^ However, the relationship between physicians’ engagement in addressing HRSNs and burnout remains unknown. Amid the ongoing health care workforce shortages and burnout,^14,15,16^ these represent critical evidence gaps within the endeavors to advance health equity while simultaneously protecting physicians’ well-being. To address these evidence gaps, we characterized physician engagement in addressing HRSNs and explored its association with burnout.

## Methods

### Study Design and Dataset

This cross-sectional study was performed using the 2022 Association of American Medical Colleges (AAMC) National Sample Survey of Physicians (NSSP),^17^ a nationally representative survey of actively practicing physicians in the US that measured a diverse array of demographic and workforce characteristics. The study was exempted by the institutional review board at the AAMC with a waiver of informed consent, as it involved secondary analysis of deidentified data. We adhered to the Strengthening the Reporting of Observational Studies in Epidemiology (STROBE) reporting guideline.^18^

The NSSP 2022 was conducted from May to November 2022. The sampling method used the following steps: (1) inclusion of all eligible respondents from NSSP 2019 and (2) replenishment of the cohort through stratified random sampling (strata were physician gender, specialty group, age group, and rural status). The University of Michigan Population Dynamics and Health Program’s recruitment strategy^17^ was used for NPPS 2022 to gather data from active physicians, some of whom were repeating respondents from NSSP 2019.

Weights were created by calibrating age group, gender, international medical graduate (IMG) status, and specialty group to align with the population distributions of the American Medical Association Physician Professional Data 2022^19^ to create nationally representative data. Further information regarding survey development, sampling, and data collection methods is documented on the AAMC Workforce Studies datasets website.^17^

### Variables

The primary independent variable was physicians’ engagement in addressing HRSNs, as measured by the NSSP item, “During the past 12 months, how often did you spend work time helping your patients meet their social needs (eg, referrals to shelters or giving vouchers for transportation)?” Engagement was measured using a 5-point ordinal scale ranging from never to daily. Respondents were also given an option to select “not certain.” Given the study’s goal of characterizing physicians’ engagement in addressing HRSNs and its association with burnout, physicians who responded “not certain” or who did not provide self-reported data for the HRSN engagement question were excluded from the analysis cohort. The distributions of “not certain” responses by physician characteristics are provided in eTable 1 in Supplement 1. To address the skewed response distribution toward lower levels of engagement, engagement levels were categorized into no engagement (never), low to moderate engagement (monthly or <1 time per month), and high engagement (weekly or daily).

The primary outcome was physician burnout, assessed by the NSSP item, “I feel burned out from my work.” Burnout was measured using a 7-point ordinal scale ranging from never to daily. This validated, nonproprietary, single-item measure of burnout was adapted from the emotional exhaustion domain of the 22-item Maslach Burnout Inventory.^20,21^ Burnout was dichotomized into low (<1 time weekly) and high (weekly or more often). The cutoff point for the dichotomization was chosen based on previous studies^22,23,24^ and concurrent validity demonstrated in the psychometric study.^25^

The demographic and clinical practice characteristics of physicians included age; self-reported gender; self-reported race and ethnicity; IMG status; specialty; practice settings (proportion in ambulatory or outpatient setting and inpatient setting); hours worked per week; engagement in teaching and/or training students, residents, or others (yes or no); use of non-English languages in patient communication (always, often, sometimes, rarely, or never); team factors (whether they routinely worked with nurse practitioners and physician assistants); and geographic locations for providing patient care (ie, high rural serving). Physicians’ gender identities were categorized into 3 groups: genderqueer or other, men or transgender men, and women or transgender women. Race and ethnicity data were categorized based on the survey’s specified options, including American Indian or Alaska Native; Asian; Black or African American; Hispanic, Latino, Latina, Latinx, or of Spanish origin; Middle Eastern or North African; Native Hawaiian or Other Pacific Islander; non-Hispanic White; multiracial (physicians who self-identified as ≥2 races); or other (participants were prompted to write in).

High rural serving was defined as 20% or more of respondents’ self-reported patient care time spent in rural areas based on the NSSP item, “What percentage of your patient care time do you spend in each of the following types of places—rural, suburban, urban?” The cutoff point of 20% was selected as it reflected a natural break in the skewed distribution of responses.^26^

Organizational factors included practice settings (private practice, system, hospital, group practice, other, or multiple settings) and the availability of behavioral health resources (access to licensed mental health professionals, referral relationships with mental health professionals, and access to nonlicensed mental health support professionals [eg, peer support, community health workers]). As a proxy for patient socioeconomic status, physicians’ reported proportions of patients’ insurance types (Medicare, Medicaid, dual eligible, commercially insured, or uninsured) were included based on the NSSP item, “What is the proportion of your patients by type of insurance?”

### Statistical Analysis

Physicians’ characteristics, including the distribution of physician engagement in addressing patients’ HRSNs, were reported using descriptive statistics. For normally distributed continuous variables, means and SDs were used, and for nonnormally distributed variables, medians and IQRs were used. Categorical variables were reported using frequencies and percentages. Missing data were not imputed.

The weighted distributions of physicians’ engagement in addressing HRSNs, by physician characteristics, were explored through bivariate analyses. χ^2^ Tests were used for categorical variables and 2-tailed *t* tests for continuous variables, with statistical tests conducted across categories. The association between physicians’ engagement in addressing HRSNs and physician burnout was examined using weighted multivariate logistic regression. The model was adjusted by the following sets of variables: demographic characteristics (eg, gender, race and ethnicity), practice-related factors (eg, specialty), organizational characteristics (eg, private vs nonprivate), and patient factors (eg, insurance type). When adding the proportion of patients with each type of insurance to the model, the variables were dichotomized using a cutoff of 20%. This cutoff was chosen to reflect a natural break in the skewed distribution of responses, indicating a meaningful distinction. The selection of variables included in the final model was guided by both statistical significance and their contextual relevance to physicians’ engagement in addressing HRSNs^27^ as well as the goodness of fit, evaluated by the Hosmer-Lemeshow test.

To explore whether the association between engagement in addressing social needs and burnout varied by specialty group, we conducted subgroup analyses based on specialty groups (primary care, surgery, emergency medicine, psychiatry, medical specialties [eg, dermatology, gastroenterology], and other [eg, radiology, physical medicine and rehabilitation]). Sensitivity analyses were performed by treating the outcome burnout as ordinal and continuous and by treating the primary independent variable HRSN engagement as a 5-point ordinal scale (1, never; 2, <1 time per month; 3, monthly; 4, weekly; 5, daily). Additionally, we conducted a sensitivity analysis including physicians who responded “not certain” for HRSN engagement (eTable 4 in Supplement 1).

Two-sided *P* < .05 was used to define statistical significance for all statistical tests. All analyses were performed using Stata, version 18.0 (StataCorp LLC).

## Results

### Physician Characteristics

Data from 5917 active physicians were gathered from NPPS 2022, among whom 2429 (41.1%) were repeating respondents from NSSP 2019. The response rate among the repeating respondents was 55.0% (2429 of 4418) and among the replenished cohort was 20.6% (3488 of 16 900) (eFigure in Supplement 1). A total of 5447 physicians were included in the analysis (Figure). The mean (SD) age of physicians was 50.9 (11.7) years. A total of 33 (0.6%) were genderqueer or other gender; 3735 (68.6%), men or transgender men; and 1679 (30.8%), women or transgender women. Ten (0.2%) were American Indian or Alaska Native; 1283 (23.6%), Asian; 135 (2.5%), Black or African American; 212 (3.9%), Hispanic, Latino, Latina, Latinx, or of Spanish origin; 122 (2.2%), Middle Eastern or North African; 13 (0.2%), Native Hawaiian or Other Pacific Islander; 3464 (63.6%), non-Hispanic White; 87 (1.6%), multiracial; and 110 (2.0%), other race and ethnicity. Less than one-quarter of the participants were IMGs (1070 [19.6%]), and nearly one-third were primary care physicians (1770 [32.5%]).

**Figure.**
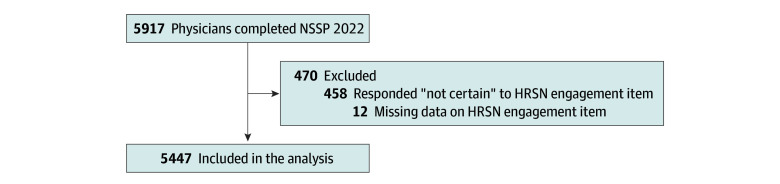
Study Cohort Engagement in health-related social need (HRSN) assessment was measured by the following item on the Association of American Medical Colleges’ National Sample Survey of Physicians (NSSP): “During the past 12 months, how often did you spend work time helping your patients meet their social needs (eg, referrals to shelters or giving vouchers for transportation)?”

The majority of physicians in this study spent most of their patient care time in ambulatory or outpatient settings (median, 85% [IQR, 25%-100%] of care time) and worked a median of 45 hours/wk (IQR, 40-55 hours/wk). Approximately one-quarter of the physicians engaged in teaching and/or research (1578 [29.0%]), and approximately half reported using a non-English language in patient communication at least some of the time (2716 [49.9%]). Among the study cohort, one-third of physicians reported high burnout (1806 [33.2%]). Physicians’ characteristics are detailed in Table 1, and their characteristics stratified by burnout are presented in eTable 2 in Supplement 1.

**Table 1.** Physician Characteristics in the Study Cohort

Characteristic	Physicians (N = 5447)^a^
**Physician factors**	
Age, y	
Mean (SD)	50.9 (11.7)
≤40	1107 (20.3)
41-50	1482 (27.2)
51-60	1386 (25.5)
≥61	1472 (27.0)
Gender identity	
Genderqueer or other	33 (0.6)
Men and transgender men	3735 (68.6)
Women and transgender women	1679 (30.8)
Race and ethnicity	
American Indian or Alaska Native	10 (0.2)
Asian	1283 (23.6)
Black or African American	135 (2.5)
Hispanic, Latino, Latina, Latinx, or of Spanish origin	212 (3.9)
Middle Eastern or North African	122 (2.2)
Native Hawaiian or Other Pacific Islander	13 (0.2)
Non-Hispanic White	3464 (63.6)
Multiracial	87 (1.6)
Other^b^	110 (2.0)
Missing data	11 (0.2)
IMG	1070 (19.6)
**Practice factors**	
Specialty	
Primary care	1770 (32.5)
Surgery	1044 (19.2)
Emergency medicine	311 (5.7)
Psychiatry	302 (5.5)
Medical specialties	1102 (20.2)
Other	918 (16.9)
Proportion of patient care in practice settings, median (IQR), %	
Ambulatory or outpatient setting	85 (25-100)
Inpatient	0 (0-25)
Missing data	67 (1.2)
Hours worked per wk	
Median (IQR)	45 (40-55)
Missing data	25 (0.5)
Engaged in teaching and/or research	1578 (29.0)
Language used in patient communication	
English only	2715 (49.9)
Moderate use of non-English language	2010 (36.9)
Frequent use of non-English language	706 (13.0)
Missing data	16 (0.3)
Frequency of work with NPs and PAs	
NPs	
Routinely	2601 (47.8)
Not routinely	2789 (51.2)
Missing data	57 (1.0)
PAs	
Routinely	2408 (44.2)
Not routinely	3011 (55.3)
Missing data	28 (0.5)
Patient care time in rural areas, %	
<20	4526 (83.1)
≥20	843 (15.5)
Missing data	78 (1.4)
**Organizational factors**	
Practice setting	
Private practice	1864 (34.2)
System	594 (10.9)
Hospital	1005 (18.5)
Group practice	841 (15.4)
Other	502 (9.2)
Multiple settings	641 (11.8)
Behavioral health resources	
Access to licensed mental health professionals	1799 (33.0)
Referral relationships with mental health professionals accessible to patients	2142 (39.3)
Access to nonlicensed mental health support professionals	455 (8.4)
**Patient-level factors**	
Insurance type, median (IQR), %	
Medicare	30 (10-40)
Medicaid	10 (3-25)
Dual eligible	2 (0-10)
Uninsured	2 (0-6)
Commercial	40 (20-57)
Missing data	23 (0.4)
Burnout	
Low	3641 (66.8)
High	1806 (33.2)

Abbreviations: IMG, international medical graduate; NP, nurse practitioner; PA, physician assistant.

^a^ Data are presented as unweighted number (percentage) of physicians unless otherwise indicated.

^b^ Participants were prompted to write a different category.

### Characteristics of Physicians’ Engagement in HRSNs

In the study cohort, 67.5% of physicians reported any engagement in addressing HRSNs, with 33.2% and 34.3% reporting low to moderate and high HRSN engagement, respectively. Variability in the distribution of physicians’ engagement in addressing HRSNs was observed across all physician-level characteristics, including demographics, use of a non-English language in patient care, and specialty (Table 2).

**Table 2.** Weighted Distributions of Physicians’ Engagement in Addressing Health-Related Social Needs, by Physician Characteristics

Physician characteristic	Engagement, %^a^			*P* value
	None	Low to moderate	High	
Overall study cohort (N = 5447)	32.4	33.2	34.3	NA
Age group, y				
≤40	28.9	26.3	44.9	<.001
41-50	28.4	32.6	39.0	
51-60	31.3	35.5	33.2	
≥61	38.8	34.7	26.5	
Gender identity				
Genderqueer or other	58.3	10.5	31.3	.004
Men and transgender men	35.1	32.7	32.2	
Women and transgender women	27.9	34.3	37.8	
Race and ethnicity				
American Indian or Alaska Native	0	97.4	2.6	.001
Asian	27.7	33.0	39.3	
Black or African American	19.9	38.7	41.4	
Hispanic, Latino, Latina, Latinx, or of Spanish origin	24.0	37.4	38.6	
Middle Eastern or North African	22.9	37.1	40.1	
Native Hawaiian or Other Pacific Islander	88.5	0.3	11.2	
Non-Hispanic White	35.5	32.6	31.9	
Multiracial	36.2	39.8	24.0	
Other^b^	11.6	39.4	49.0	
IMG status				
Non-IMG	35.4	33.2	31.4	<.001
IMG	23.0	33.4	43.7	
Specialty				
Primary care	23.3	37.7	39.1	<.001
Surgery	42.6	37.3	20.1	
Emergency medicine	9.8	19.0	71.1	
Psychiatry	23.4	31.7	44.9	
Medical specialties	30.8	38.0	31.2	
Other	48.5	24.1	27.3	
Engaged in teaching and/or research				
No	35.5	33.1	31.4	<.001
Yes	24.4	33.6	42.1	
Language used in patient communication				
English only	36.6	33.6	29.7	<.001
Moderate use of non-English language	29.4	34.1	36.5	
Frequent use of non-English language	25.4	29.4	45.2	
Patient care time in rural areas, %				
<20	34.2	33.2	32.7	<.001
≥20	21.6	32.9	45.5	
Proportion of patients with Medicare, %				
<20	32.4	33.4	34.2	.10
≥20	32.5	33.2	34.4	
Proportion of patients with Medicaid and/or dual coverage, %				
<20	38.7	37.1	24.3	<.001
≥20	24.0	28.1	48.0	
Proportion of uninsured patients				
<20	32.8	33.4	33.8	.04
≥20	23.9	28.8	47.3	

Abbreviations: IMG, international medical graduate; NA, not applicable.

^a^ Weighted percentages are presented as row percentages within each category.

^b^ Participants were prompted to write a different category.

Physicians aged 40 years or younger exhibited the highest percentage (44.9%) reporting high engagement in addressing HRSNs compared with those aged 41 to 50 years (39.0%), 51 to 60 years (33.2%), and 61 years or older (26.5%) (*P* < .001). Women and transgender women physicians reported higher HRSN engagement (37.8%) compared with genderqueer or other physicians (31.3%) and men and transgender men (32.2%) (*P* = .004). Additionally, physicians identifying as Black or African American (41.4%) or as other race and ethnicity (49.0%) had the greatest proportions reporting high engagement (*P* < .001).

A greater percentage of IMG physicians reported high engagement in addressing HRSNs compared with their counterparts (43.7% vs 31.4%; *P* < .001). Among specialty groups, physicians in emergency medicine (71.1%), psychiatry (44.9%), and primary care (39.1%) most commonly reported high engagement in addressing HRSNs (*P* < .001). Physicians who frequently communicated with their patients using non-English languages reported higher HRSN engagement (45.2%) than those who used non-English languages less frequently (36.5%) or who only used English (29.7%) (*P* < .001).

### Association Between HRSN Engagement and Burnout

Compared with no HRSN engagement, low to moderate (adjusted odds ratio [AOR], 1.33; 95% CI, 1.03-1.72; *P* = .03) and high (AOR, 1.72; 95% CI, 1.39-2.27; *P* < .001) HRSN engagement were significantly associated with high burnout (Table 3). We also ran the model with the same set of variables among each specialty group (Table 4). While there was no association between HRSN engagement and burnout among physicians in emergency medicine, psychiatry, and medical specialties, high vs no HRSN engagement was associated with high burnout among physicians in primary care (AOR, 1.89; 95% CI, 1.26-2.84; *P* = .002), surgical specialties (AOR, 3.40; 95% CI, 1.67-6.93; *P* = .001), and other specialties (AOR, 2.21; 95% CI, 1.11-4.37; *P* = .02). Sensitivity analyses showed a pattern in the association between HRSN engagement and burnout that was consistent with the main model (eTable 4 in Supplement 1).

**Table 3.** Multivariate Logistic Regression Analysis of the Association Between Physicians’ Engagement in Addressing Health-Related Social Needs and Burnout^a^

Variable	AOR (95% CI)^b^	*P* value
Engagement in HRSNs		
None	1 [Reference]	NA
Low to moderate	1.33 (1.03-1.72)	.03
High	1.72 (1.39-2.27)	<.001
Age, y		
≤40	1 [Reference]	NA
41-50	1.14 (0.80-1.64)	.45
51-60	1.00 (0.70-1.43)	.98
≥61	0.59 (0.41-0.86)	.005
Gender identity		
Genderqueer or other	3.32 (0.74-14.94)	.12
Men and transgender men	1 [Reference]	NA
Women and transgender women	1.04 (0.83-1.30)	.72
Race and ethnicity		
American Indian or Alaska Native	1.48 (0.13-16.51)	.75
Asian	0.74 (0.58-0.96)	.02
Black or African American	0.74 (0.35-1.55)	.42
Hispanic, Latino, Latina, Latinx, or of Spanish origin	0.50 (0.27-0.90)	.02
Middle Eastern or North African	1.42 (0.84-2.39)	.19
Native Hawaiian or Other Pacific Islander	0.03 (0.01-0.11)	<.001
Non-Hispanic White	1 [Reference]	NA
Multiracial	0.73 (0.28-1.90)	.52
Other^c^	0.82 (0.41-1.64)	.57
IMG status		
Non-IMG	1 [Reference]	NA
IMG	0.74 (0.56-0.97)	.03
Specialty		
Medical specialties	1 [Reference]	NA
Primary care	1.22 (0.92-1.62)	.17
Surgery	1.04 (0.75-1.44)	.83
Emergency medicine	1.39 (0.84-2.30)	.20
Psychiatry	1.12 (0.69-1.82)	.64
Other	0.82 (0.57-1.17)	.27
Teaching and/or research		
No engagement	1 [Reference]	NA
Engagement	0.90 (0.71-1.15)	.42
Patient care time in rural areas, %		
<20	1 [Reference]	NA
≥20	1.06 (0.81-1.40)	.65
Practice setting		
Private practice	1 [Reference]	NA
System	1.34 (0.96-1.87)	.09
Hospital	1.28 (0.93-1.76)	.13
Group practice	1.47 (1.09-1.98)	.01
Other	0.96 (0.66-1.40)	.84
Multiple settings	0.86 (0.59-1.25)	.42
Patients’ insurance type		
<20% Medicaid and/or dual coverage	1 [Reference]	NA
≥20% Medicaid and/or dual coverage	0.80 (0.65-1.00)	.05
<20% Uninsured	1 [Reference]	NA
≥20% Uninsured	1.24 (0.75-2.04)	.40

Abbreviations: AOR, adjusted odds ratio; HRSN, health-related social needs; IMG, international medical gradute; NA, not applicable.

^a^ N = 5358. A total of 89 respondents had missing data for 1 or more of the following variables: race and ethnicity, patient care time in rural areas, or patient insurance type.

^b^ Adjusted for demographic characteristics (eg, gender, race and ethnicity), practice-related factors (eg, specialty), organizational characteristics (eg, private vs nonprivate), and patient factors (eg, insurance type).

^c^ Participants were prompted to write a different category.

**Table 4.** Subgroup Analysis of Physician Engagement in Addressing HRSNs and Its Association With Burnout, by Specialty Group^a^

Specialty group	AOR (95% CI)	*P* value
All physicians (N = 5378)		
Low to moderate	1.33 (1.03-1.72)	.03
High	1.72 (1.39-2.27)	<.001
Primary care (n = 1733)		
Low to moderate	1.32 (0.89-1.97)	.16
High	1.89 (1.26-2.84)	.002
Surgery (n = 1029)		
Low to moderate	1.93 (1.12-3.34)	.02
High	3.40 (1.67-6.93)	.001
Emergency medicine (n = 307)		
Low to moderate	1.05 (0.12-9.27)	.96
High	0.45 (0.06-3.31)	.43
Psychiatry (n = 293)		
Low to moderate	0.59 (0.13-2.31)	.49
High	0.50 (0.13-1.88)	.30
Medical specialties (n = 1081)		
Low to moderate	1.46 (0.81-2.62)	.20
High	1.35 (0.72-2.52)	.35
Other (n = 903)		
Low to moderate	0.97 (0.47-2.00)	.94
High	2.21 (1.11-4.37)	.02

Abbreviations: AOR, adjusted odds ratio; HRSNs, health-related social needs.

^a^ Analysis was controlled for variables included in the same multivariate logistic regression model as in Table 3. The reference category was no HRSN engagement. The full regression results are included in eTable 3 in Supplement 1.

## Discussion

The nationally representative sample of physicians in this study revealed that 34.3% spent their patient care time addressing HRSNs on a regular basis (ie, weekly or more frequently). In recent decades, the health care sector has taken an active role in addressing social drivers of health (SDOHs) by collecting patients’ HRSN information, such as through screenings, and collaborating with community and social services agencies for intervention purposes.^10,28^ In January 2024, the CMS introduced a new Healthcare Common Procedure Coding System Level II payment code, G0136, reimbursing physicians to administer an SDOH risk assessment no more than once every 6 months. This new code supports the transition from simple screening to comprehensive assessment, followed by appropriate follow-up or referrals.^9^ With the introduction of this new payment provision, there is a plausible expectation that physicians’ engagement in addressing HRSNs will increase.

The findings of this study suggest that physicians from historically marginalized communities are more frequently addressing HRSNs. Specifically, high engagement in addressing HRSNs was observed among physicians identifying as women or transgender women, those reporting Black or African American or other race and ethnicity, IMGs, and those who frequently used non-English languages in patient communication. From the perspective of race-conscious professionalism,^29,30^ this could possibly be explained by intrinsic factors, with physicians from certain racial and ethnic groups potentially feeling a stronger commitment to addressing HRSNs. Importantly, these findings add an additional layer to diversity, equity, and inclusion efforts in medicine by critically considering the “minority tax”—the extra responsibilities that historically marginalized physicians often experience.^31,32^

Our findings showed that higher engagement in addressing HRSNs was associated with a high rate of burnout. Subgroup analysis showed that while a similar association was observed among physicians in primary care, surgical specialties, and other specialties, there was no such association among physicians in emergency medicine, psychiatry, and medical specialties. One potential explanation could be the emphasis placed on addressing HRSNs within the residency training in emergency medicine and psychiatry; thus, the effect of HRSN engagement on burnout may not be substantial in these specialties, as the physicians are accustomed to engaging in this type of work.^33,34^

The findings from this study complement prior work examining the association between patients’ HRSNs and clinicians’ well-being.^11,12,13^ From the perspective of organizational capacity, studies have demonstrated that having fewer organizational resources to address patients’ social needs is associated with higher levels of clinician burnout.^11,13^ In a mixed-methods study exploring pathways linking organizational resources and clinician burnout, 3 potential pathways were identified: the association of unmet social needs with the effectiveness and efficacy of medical care, the integration of social services into medical services to support clinicians in focusing on their medical role, and health care sector and community-level structural barriers negatively affecting clinicians’ burnout.^12^ Building on this growing body of work, our findings suggest that higher levels of individual physician engagement in addressing HSRNs may negatively contribute to physician burnout. One possible explanation for the association between increased engagement in addressing HRSNs and greater burnout is that patients’ HRSNs are complex and often cannot be resolved through 1-time efforts in clinical settings but require sustained, long-term collective efforts across care teams and sectors. Recognizing patients’ ongoing, unmet HRSNs without being able to fully address them could potentially lead to a sense of helplessness, contributing to burnout.^12^ Addressing HRSNs necessitates interdisciplinary teamwork, such as HRSN screening often being led by nonphysician staff (eg, nurses, social workers, and community health workers)^35^; therefore, training and education can be incorporated to help physicians effectively collaborate with interprofessional team members to address HRSNs for patient populations.

Furthermore, as physicians respond to the consequences of inequities in health care and other factors on patients’ health by addressing HRSNs and as their time for this work becomes acknowledged in claims payment systems, it is critical to increase parallel efforts in policy and organizational investment to adequately support physicians in this work. Various interventions have been implemented to address HRSNs, such as cross-sector collaboration between health care organizations and community-based organizations (eg, supplemental nutrition assistance programs and housing services)^36^ and establishing an information technology (IT) infrastructure to integrate a social services resources directory into electronic health records to help patients connect with resources.^37^ Understanding of whether these interventions can actually help alleviate burden associated with addressing HRSNs is needed, allowing physicians to focus more on their medical role.^38^

### Limitations

This study has several limitations. First, due to the cross-sectional nature of the study design, we were unable to examine a causal relationship between physicians’ engagement in addressing social needs and burnout. Second, because physician-reported frequency of addressing HRSNs based on the NPPS item was used as a proxy for their engagement, the findings may not accurately reflect physicians’ involvement in addressing HRSNs. Third, in our study, burnout was measured using a single item derived from the emotional exhaustion domain of the Maslach Burnout Inventory and treated as a dichotomized variable of high vs low burnout, which may not fully capture the multidimensional nature of burnout as assessed by the complete inventory.^39,40^

Our study sheds light on 1 dimension of the association between physician engagement with HRSNs and burnout. In actuality, this is a more intricate and dynamic relationship shaped by various contextual and individual factors (eg, organizational infrastructure, clinical flows, and intrinsic motivation).^11,12,13,41,42^ Future studies using qualitative research and/or mixed-methods approaches could provide a more in-depth understanding of contextual factors, such as organizational structures, clinical flows, and IT infrastructure, that shape physicians’ experiences in addressing HRSNs and the relationship between physicians’ engagement in addressing HRSNs and burnout.

## Conclusions

In this cross-sectional study of 5447 nationally representative physicians in the US, we identified variability in physicians’ engagement in addressing HRSNs and found that a higher degree of engagement was associated with burnout. As growing numbers of health equity strategies are developed and implemented, our study findings offer insight into strategies for mitigating physician burnout and suggest the need for a thorough assessment of the potential unintended consequences of physicians’ engagement in addressing HRSNs on their well-being.

## Notes

Supplement 1. **eFigure.** Flow Diagram of NSSP 2022 Respondents **eTable 1.** Characteristics of Physicians With “Not Certain” Response in the HRSN Engagement Item **eTable 2.** Physicians’ Characteristics by Burnout **eTable 3.** Multivariate Logistic Regression Analysis of Association Between Physicians’ Engagement in Addressing Health-Related Social Needs and Burnout, by Specialty **eTable 4.** Sensitivity Analyses—Multivariate Ordinary Least Squares Regression Analysis of Association Between Physicians’ Engagement in Addressing Health-Related Social Needs and Burnout

Supplement 2.
Data Sharing Statement
