# Supplementary material for: Physician Engagement in Addressing Health-Related Social Needs and Burnout
Source: JAMA Netw Open. 2024 Dec 30;7(12):e2452152. doi: 10.1001/jamanetworkopen.2024.52152 (PMC11686412; doi:10.1001/jamanetworkopen.2024.52152)
Supplement: Supplement 1. — eFigure. Flow Diagram of NSSP 2022 Respondents eTable 1. Characteristics of Physicians With “Not Certain” Response in the HRSN Engagement Item eTable 2. Physicians’ Characteristics by Burnout eTable 3. Multivariate Logistic Regression Analysis of Association Between Physicians’ Engagement in Addressing Health-Related Social Needs and Burnout, by Specialty eTable 4. Sensitivity Analyses—Multivariate Ordinary Least Squares Regression Analysis of Association Between Physicians’ Engagement in Addressing Health-Related Social Needs and Burnout [file jamanetwopen-e2452152-s001.pdf]

Supplementary Online Content

Tabata-Kelly M, Hu X, Dill MJ, et al. Physician engagement in addressing health-related social needs and burnout. *JAMA Netw Open*. 2024;7(12):e2452152. doi:10.1001/jamanetworkopen.2024.52152

eFigure. Flow Diagram of NSSP 2022 Respondents
eTable 1. Characteristics of Physicians With "Not Certain" Response in the HRSN Engagement Item
eTable 2. Physicians' Characteristics by Burnout
eTable 3. Multivariate Logistic Regression Analysis of Association Between Physicians' Engagement in Addressing Health-Related Social Needs and Burnout, by Specialty
eTable 4. Sensitivity Analyses—Multivariate Ordinary Least Squares Regression Analysis of Association Between Physicians' Engagement in Addressing Health-Related Social Needs and Burnout

This supplementary material has been provided by the authors to give readers additional information about their work.

© 2024 Tabata-Kelly M et al. *JAMA Network Open*.

eFigure: Flow diagram for NSSP 2022 respondents

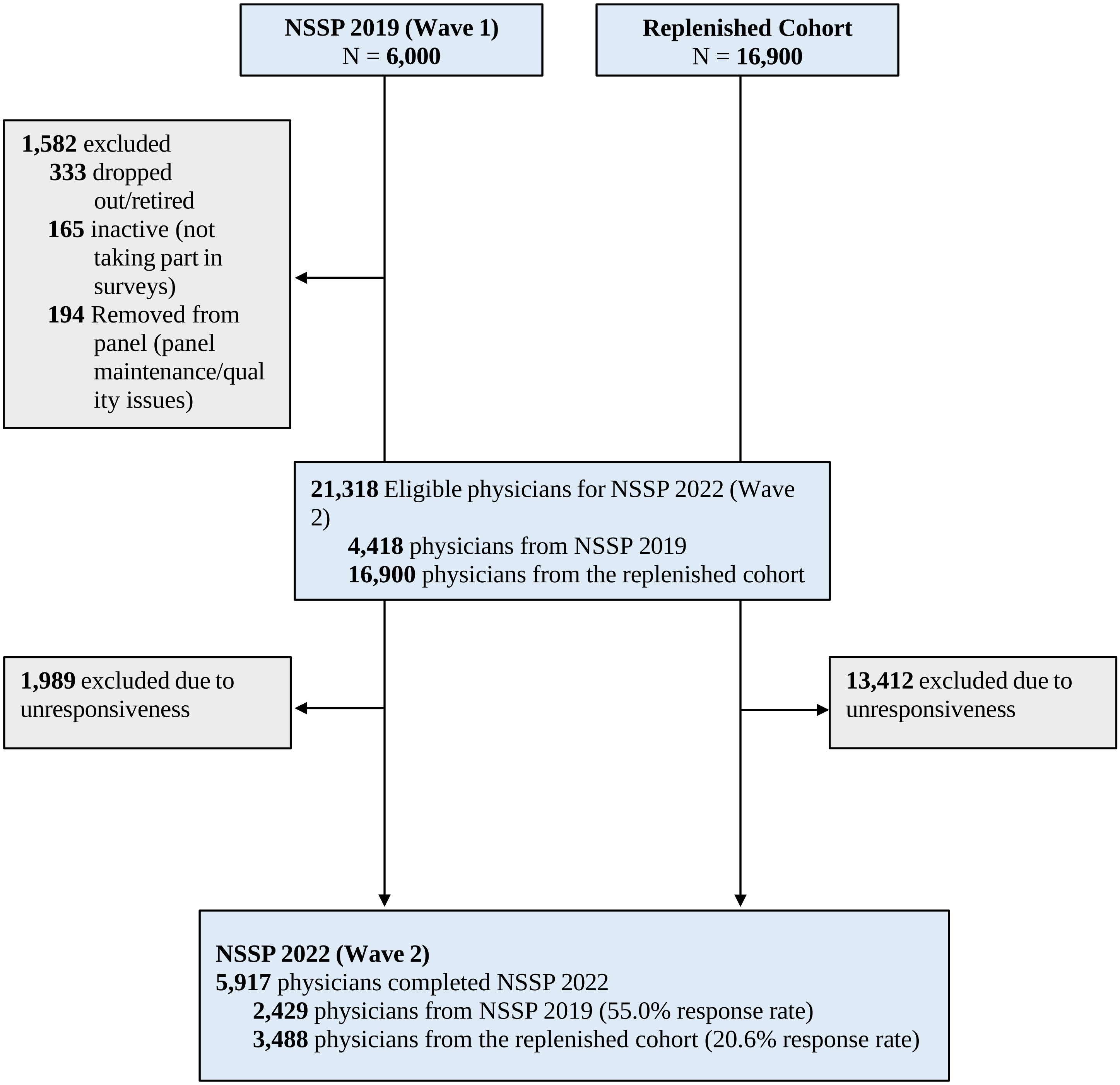

© 2024 Tabata-Kelly M et al. *JAMA Network Open*.

eTable 1: Characteristics of Physicians with "Not Certain" Response in the HRSN Engagement Item*

Characteristics	Physicians with "Not Certain" Response in the HRSN Engagement Item (N=458)
**Age**	
40 and younger	80 (17.47)
41-50	111 (24.24)
51-60	132 (28.82)
61 and older	135 (29.48)
**Gender identity**	
Woman and Trans women	106 (23.14)
Man and Trans Man	345 (75.33)
Genderqueer or other	7 (1.53)
**Race/Ethnicity(a)**	
American Indian or Alaskan Native	1 (0.22)
Asian	128 (27.95)
Black or African American	9 (1.97)
Native Hawaiian or other Pacific Islander	0 (0.00)
Non-Hispanic White	252 (55.02)
Middle eastern or North African	17 (3.71)
Multi racial	5 (1.09)
Other	15 (3.28)
Hispanic, Latino or Spanish origin	26 (5.68)
Missing data	5 (1.09)
**International Medical Graduates (IMGs)**	
Non-IMG	309 (67.47)
IMG	149 (32.53)
**Specialty**	
Primary care	130 (28.38)
Surgery	97 (21.18)
Emergency medicine	11 (2.40)
Psychiatry	24 (5.24)
Medical Specialties	128 (27.95)
Other	68 (14.85)
**Practice settings**	
Private practice	191 (41.70)
System	52 (11.35)
Hospital	70 (15.28)
Group practice	58 (12.66)
Other	42 (9.17)
Multiple settings	45 (9.83)

* The NSSP item: "*During the past 12 months, how often did you spend work time helping your patients meet their social needs (e.g., referrals to shelters or giving vouchers for transportation)?*"

© 2024 Tabata-Kelly M et al. *JAMA Network Open*.

eTable 2. Physicians' Characteristics by Burnout (Unweighted, N=5,447)

Characteristics	Overall (N=5,447)	Low burnout (N=3,641)	High burnout (N= 1,806)	P value
Physician factors				
**Age**				
40 and younger	1,107 (20.32)	712 (19.56)	395 (21.87)	<0.001
41-50	1,482 (27.21)	913 (25.08)	569 (31.51)	
51-60	1,386 (25.45)	905 (25.08)	569 (31.51)	
61 and older	1,472 (27.02)	1,111 (30.51)	361 (19.99)	
**Gender identity**				
Woman and Trans women	1,679 (30.82)	1,017 (27.93)	662 (36.66)	<0.001
Man and Trans Man	3,735 (68.57)	2,603 (71.49)	1,132 (62.68)	
Genderqueer or other	33 (0.61)	21 (0.58)	12 (0.66)	
**Race/Ethnicity(a)**				
American Indian or Alaskan Native	10 (0.18)	3 (0.08)	7 (0.39)	0.02
Asian	1,283 (23.55)	902 (24.77)	381 (21.10)	
Black or African American	135 (2.48)	92 (2.53)	43 (2.39)	
Native Hawaiian or other Pacific Islander	13 (0.24)	7 (0.19)	6 (0.33)	
Non-Hispanic White	3,464 (63.59)	2,287 (62.81)	1,177 (65.17)	
Middle eastern or North African	122 (2.24)	75 (2.06)	47 (2.60)	
Multi racial	87 (1.60)	57 (1.57)	30 (1.66)	
Other	110 (2.02)	68 (1.87)	42 (2.33)	
Hispanic, Latino or Spanish origin	212 (3.89)	144 (3.95)	68 (3.77)	
Missing data	11 (0.20)	6 (0.16)	5 (0.28)	
**International Medical Graduates (IMGs)**				
IMG	1,070 (19.64)	762 (20.93)	308 (17.05)	0.001
Practice factors				
**Specialty**				
Primary care	1,770 (32.49)	1,106 (30.38)	664 (36.77)	<0.001
Surgery	1,044 (19.17)	741 (20.35)	303 (16.78)	
Emergency medicine	311 (5.71)	179 (4.92)	132 (7.31)	
Psychiatry	302 (5.54)	202 (5.55)	100 (5.54)	
Medical Specialties	1,102 (20.23)	769 (21.12)	333 (18.44)	
Other	918 (16.85)	644 (17.69)	274 (15.17)	
**Patient care % in practice settings (Median [IQR])**				
Ambulatory or Outpatient setting	85% (25-100)	85% (30-100)	88% (25-100)	0.85
Inpatient	0% (0-25)	2% (0-30)	0% (0-25)	0.001
Missing data	67 (1.23)	45 (1.24)	22 (1.22)	
**Hours worked per week**				
Median (IQR)	45 (40-55)	45 (38-55)	50 (40-60)	<0.001
Missing data	25 (0.46)	19 (0.52)	6 (0.33)	
**Engage in teaching and/or research**				

© 2024 Tabata-Kelly M et al. *JAMA Network Open*.

Characteristics	Overall (N=5,447)	Low burnout (N=3,641)	High burnout (N= 1,806)	P value
Yes	1,578 (28.97)	1,110 (30.49)	468 (25.91)	<0.001
**Non-English language use in patient communication**				
English only	2,715 (49.99)	1,794 (49.27)	921 (51.00)	0.03
Moderate use	2,010 (37.01)	1,337 (36.72)	673 (37.26)	
Frequent use	706 (13.00)	503 (13.81)	203 (11.24)	
Missing data	16 (0.29)	7 (0.19)	9 (0.50)	
**Nurse practitioner and physician assistants**				
Routinely work with NP	2,601 (47.75)	1,687 (46.33)	914 (50.61)	0.004
Not routinely work with NP	2,789 (51.20)	1,912 (52.51)	877 (48.56)	
Missing data	57 (1.05)	42 (1.15)	15 (0.83)	
Routinely work with PA	2,408 (44.21)	1,584 (43.50)	824 (45.63)	0.15
Not routinely work with PA	3,011 (55.28)	2,037 (55.95)	974 (53.93)	
Missing data	28 (0.51)	20 (0.55)	8 (0.44)	
**Geographical locations for providing patient care**				
Rual serving (<20%)	4,526 (83.09)	3,060 (84.04)	1,466 (81.17)	0.007
Rural serving (≥20%)	843 (15.48)	530 (14.56)	313 (17.33)	
Missing data	78 (1.43)	51 (1.40)	27 (1.50)	
Organizational factors				
**Practice settings**				
Private practice	1,864 (34.22)	1,294 (35.54)	570 (31.56)	<0.001
System	594 (10.91)	353 (9.70)	241 (13.34)	
Hospital	1,005 (18.45)	660 (18.13)	345 (19.10)	
Group practice	841 (15.44)	551 (15.13)	290 (16.06)	
Other	502 (9.22)	342 (9.39)	160 (8.86)	
Multiple settings	641 (11.77)	441 (12.11)	200 (11.07)	
**Behavioral health resources**				
Access to Licensed mental health providers	1,799 (33.03)	1,177 (32.33)	622 (34.44)	0.12
Referral relationship(s) with mental health providers who are accessible to your patient	2,142 (39.32)	1,428 (39.22)	714 (39.53)	0.65
Access to Non-licensed mental health support providers	455 (8.35)	300 (8.24)	155 (8.58)	0.67
Patient-level factors				
**Patients' insurance type**				
Medicare (Median [IQR])	30% (10-40)	30% (10-40)	30% (10-40)	0.82
Medicaid (Median [IQR])	10% (3-25)	10% (3-25)	10% (3-25)	0.17
Dual (Median [IQR])	2% (0-10)	2% (0-10)	3% (0-1)	0.83
Uninsured (Median [IQR])	2% (0-6)	2% (0-5)	3% (0-10)	0.006
Commercial (Median [IQR])	40 % (20-57)	40% (20-55)	40% (20-58)	0.59
Missing data	23 (0.42)	14 (0.38)	9 (0.50)	

© 2024 Tabata-Kelly M et al. *JAMA Network Open*.

eTable 3. Multivariate Logistic Regression Analysis of the Association between Physicians' Engagement in Addressing Health-Related Social Needs and Burnout by Specialty

***A. Primary Care* (n =1,733)**

Variables	AOR (95% CI)	P value
**Engagement in HRSN**		
No engagement	Ref.	Ref.
Low-moderate engagement	1.32 (0.89-1.97)	0.164
High engagement	1.89 (1.26-2.84)	0.002
**Age**		
40 and younger	Ref.	Ref.
41-50	0.99 (0.51-1.63)	0.748
51-60	0.87 (0.50-1.54)	0.643
61 and older	0.61 (0.35-1.06)	0.080
**Gender identity**		
Man and Trans man	Ref.	Ref.
Woman and Trans woman	0.89 (0.65-1.22)	0.465
Genderqueer or other	3.18 (0.21-49.09)	0.408
**Race/Ethnicity**		
Non-Hispanic White	Ref.	Ref.
Hispanic, Latino or Spanish origin	0.86 (0.36-2.04)	0.730
American Indian or Alaskan Native	1.45 (0.12-18.23)	0.773
Asian	0.72 (0.48-1.08)	0.112
Black or African American	0.49 (0.18-1.33)	0.162
Native Hawaiian or other Pacific Islander	0.01 (0.00-0.07)	p<0.001
Middle eastern or North African	0.59 (0.22-1.56)	0.290
Multi racial	0.75 (0.17-3.43)	0.715
Other	0.57 (0.17-1.92)	0.366
**International Medical Graduates (IMGs)**		
Non-IMG	Ref.	Ref.
IMG	0.69 (0.46-1.03)	0.070
**Engage in teaching and/or research**		
No teaching/research engagement	Ref.	Ref.
Teaching/research engagement	0.74 (0.48-1.15)	0.179
**Geographical locations for providing patient care**		
Rural serving (<20%)	Ref.	Ref.
High rural serving (>=20%)	0.95 (0.63-1.42)	0.789
**Practice settings**		
Private practice	Ref.	Ref.
System	1.61 (1.03-2.51)	0.035
Hospital	1.32 (0.73-2.39)	0.353
Group practice	1.19 (0.76-1.85)	0.451
Other	1.37 (0.76-2.46)	0.294
Multiple settings	0.92 (0.51-1.69)	0.799

© 2024 Tabata-Kelly M et al. *JAMA Network Open*.

**Patients' insurance type**		
Proportion of Medicaid and/or dual coverage patients (<20%)*	Ref.	Ref.
Proportion of Medicaid and/or dual coverage patients (>=20%)	0.73 (0.52-1.02)	0.063
Proportion of uninsured patients (<20%)	Ref.	Ref.
Proportion of uninsured patients (>=20%)	0.78 (0.32-1.90)	0.579

B. Surgery (n =1,029)

Variables	AOR (95% CI)	P value
**Engagement in HRSN**		
No engagement	Ref.	Ref.
Low-moderate engagement	1.93 (1.12-3.34)	0.018
High engagement	3.40 (1.67-6.93)	0.001
**Age**		
40 and younger	Ref.	Ref.
41-50	2.83 (0.90-8.84)	0.074
51-60	3.40 (1.12-10,31)	0.031
61 and older	2.15 (0.70-6.62)	0.179
**Gender identity**		
Man and Trans man	Ref.	Ref.
Woman and Trans woman	1.18 (0.67-2.07)	0.571
Genderqueer or other	-*	-*
**Race/Ethnicity**		
Non-Hispanic White	Ref.	Ref.
Hispanic, Latino or Spanish origin	0.02 (0.01-0.06)	p<0.001
American Indian or Alaskan Native	Omitted**	Omitted**
Asian	0.46 (0.22-0.95)	0.035
Black or African American	0.33 (0.03-3.58)	0.363
Native Hawaiian or other Pacific Islander	-*	-*
Middle eastern or North African	3.77 (0.91-15.67)	0.067
Multi racial	1.29 (0.16-10.64)	0.814
Other	3.16 (0.52-19.35)	0.214
**International Medical Graduates (IMGs)**		
Non-IMG	Ref.	Ref.
IMG	0.49 (0.20-1.18)	0.112
**Engage in teaching and/or research**		
No teaching/research engagement	Ref.	Ref.
Teaching/research engagement	1.10 (0.63-1.95)	0.735
**Geographical locations for providing patient care**		
Rural serving (<20%)	Ref.	Ref.
High rural serving (>=20%)	0.97 (0.46-2.04)	0.938
**Practice settings**		
Private practice	Ref.	Ref.

© 2024 Tabata-Kelly M et al. *JAMA Network Open*.

System	2.98 (1.06-8.39)	0.039
Hospital	1.96 (0.95-4.05)	0.069
Group practice	3.65 (1.69-7.87)	0.001
Other	1.64 (0.60-4.43)	0.334
Multiple settings	0.74 (0.32-1.69)	0.474
**Patients' insurance type**		
Proportion of Medicaid and/or dual coverage patients (<20%)*	Ref.	Ref.
Proportion of Medicaid and/or dual coverage patients (>=20%)	0.59 (0.34-1.02)	0.061
Proportion of uninsured patients (<20%)	Ref.	Ref.
Proportion of uninsured patients (>=20%)	0.92 (0.38-2.18)	0.842

***The variable was omitted due to perfect prediction.
**The variable was omitted due to the small sample size.

C. Emergency Medicine (n =307)

Variables	AOR (95% CI)	P value
**Engagement in HRSN**		
No engagement	Ref.	Ref.
Low-moderate engagement	1.05 (0.12-9.27)	0.962
High engagement	0.45 (0.06-3.31)	0.432
**Age**		
40 and younger	Ref.	Ref.
41-50	0.62 (0.13-3.08)	0.560
51-60	0.901 (0.18-4.42)	0.898
61 and older	0.05 (0.01-0.42)	0.006
**Gender identity**		
Man and Trans man	Ref.	Ref.
Woman and Trans woman	3.93 (1.21-12.75)	0.023
Genderqueer or other	-*	-*
**Race/Ethnicity**		
Non-Hispanic White	Ref.	Ref.
Hispanic, Latino or Spanish origin	8.99 (0.54-150.91)	0.127
American Indian or Alaskan Native	Omitted**	Omitted**
Asian	2.82 (0.54-14.86)	0.221
Black or African American	0.01 (0.00-0.11)	p<0.001
Native Hawaiian or other Pacific Islander	Omitted**	Omitted**
Middle eastern or North African	0.33 (0.03-3.06)	0.326
Multi racial	0.32 (0.00-64.76)	0.677
Other	0.02 (0.00-0.37)	0.009
**International Medical Graduates (IMGs)**		
Non-IMG	Ref.	Ref.
IMG	1.02 (0.08-13.19)	0.986
**Engage in teaching and/or research**		

© 2024 Tabata-Kelly M et al. *JAMA Network Open*.

No teaching/research engagement	Ref.	Ref.
Teaching/research engagement	2.52 (0.71-8.91)	0.150
**Geographical locations for providing patient care**		
Rural serving (<20%)	Ref.	Ref.
High rural serving (>=20%)	2.92 (0.79-10.76)	0.106
**Practice settings**		
Private practice	Ref.	Ref.
System	0.70 (0.07-7.19)	0.764
Hospital	1.49 (0.26-8.51)	0.652
Group practice	0.81 (0.14-4.70)	0.819
Other	1.16 (0.14-9.79)	0.893
Multiple settings	0.11 (0.01-1.10)	0.060
**Patients' insurance type**		
Proportion of Medicaid and/or dual coverage patients (<20%)*	Ref.	Ref.
Proportion of Medicaid and/or dual coverage patients (>=20%)	0.29 (0.75-1.12)	0.073
Proportion of uninsured patients (<20%)	Ref.	Ref.
Proportion of uninsured patients (>=20%)	12.08 (2.77-52.72)	0.001

***The variable was omitted due to perfect prediction.
**The variable was omitted due to the small sample size.

D. Psychiatry (n =293)

Variables	AOR (95% CI)	P value
**Engagement in HRSN**		
No engagement	Ref.	Ref.
Low-moderate engagement	0.59 (0.13-2.71)	0.494
High engagement	0.50 (0.13-1.88)	0.304
**Age**		
40 and younger	Ref.	Ref.
41-50	3.40 (0.33-35.25)	0.305
51-60	1.67 (0.18-15.11)	0.648
61 and older	0.79 (0.07-8.58)	0.847
**Gender identity**		
Man and Trans man	Ref.	Ref.
Woman and Trans woman	1.07 (0.37-3.13)	0.902
Genderqueer or other	0.02 (0.00-1.87)	0.093
**Race/Ethnicity**		
Non-Hispanic White	Ref.	Ref.
Hispanic, Latino or Spanish origin	0.10 (0.01-1.06)	0.056
American Indian or Alaskan Native	Omitted**	Omitted**
Asian	0.20 (0.04-0.92)	0.039
Black or African American	2.06 (0.18-23.0)	0.558
Native Hawaiian or other Pacific Islander	Omitted**	Omitted**

© 2024 Tabata-Kelly M et al. *JAMA Network Open*.

Middle eastern or North African	-*	-*
Multi racial	0.07 (0.00-2.35)	0.139
Other	75.22 (4.04-1401.13)	0.004
**International Medical Graduates (IMGs)**		
Non-IMG	Ref.	Ref.
IMG	1.22 (0.36-4.19)	0.751
**Engage in teaching and/or research**		
No teaching/research engagement	Ref.	Ref.
Teaching/research engagement	0.19 (0.05-0.70)	0.012
**Geographical locations for providing patient care**		
Rural serving (<20%)	Ref.	Ref.
High rural serving (>=20%)	1.92 (0.51-7.16)	0.333
**Practice settings**		
Private practice	Ref.	Ref.
System	0.94 (0.20-4.43)	0.938
Hospital	7.12 (1.15-44.03)	0.035
Group practice	2.49 (0.43-13.96)	0.313
Other	1.54 (0.35-6.38)	0.570
Multiple settings	1.09 (0.11-10.71)	0.940
**Patients' insurance type**		
Proportion of Medicaid and/or dual coverage patients (<20%)*	Ref.	Ref.
Proportion of Medicaid and/or dual coverage patients (>=20%)	1.88 (0.51-6.88)	0.342
Proportion of uninsured patients (<20%)	Ref.	Ref.
Proportion of uninsured patients (>=20%)	43.33 (4.41 - 425.33)	0.001

*****The variable was omitted due to perfect prediction.
**The variable was omitted due to the small sample size.

E. Medical Specialties (n =1,081)

Variables	AOR (95% CI)	P value
**Engagement in HRSN**		
No engagement	Ref.	Ref.
Low-moderate engagement	1.46 (0.81-2.62)	0.204
High engagement	1.35 (0.72-2.52)	0.346
**Age**		
40 and younger	Ref.	Ref.
41-50	1.24 (0.51-3.00)	0.631
51-60	0.82 (0.33-2.02)	0.665
61 and older	0.44 (0.17-1.11)	0.082
**Gender identity**		
Man and Trans man	Ref.	Ref.
Woman and Trans woman	0.92 (0.54-1.58)	0.763

© 2024 Tabata-Kelly M et al. *JAMA Network Open*.

Genderqueer or other	0.08 (0.01-0.74)	0.026
**Race/Ethnicity**		
Non-Hispanic White	Ref.	Ref.
Hispanic, Latino or Spanish origin	0.47 (0.13-1.72)	0.252
American Indian or Alaskan Native	-*	-*
Asian	0.99 (0.57-1.71)	0.970
Black or African American	0.63 (0.10-3.75)	0.610
Native Hawaiian or other Pacific Islander	0.02 (0.00-0.45)	0.012
Middle eastern or North African	2.12 (0.77-5.80)	0.144
Multi racial	0.08 (0.01-0.54)	0.010
Other	0.96 (0.28-3.23)	0.943
**International Medical Graduates (IMGs)**		
Non-IMG	Ref.	Ref.
IMG	0.75 (0.43-1.31)	0.317
**Engage in teaching and/or research**		
No teaching/research engagement	Ref.	Ref.
Teaching/research engagement	0.97 (0.56-1.67)	0.912
**Geographical locations for providing patient care**		
Rural serving (<20%)	Ref.	Ref.
High rural serving (>=20%)	1.25 (0.59-2.67)	0.562
**Practice settings**		
Private practice	Ref.	Ref.
System	0.72 (0.32-1.63)	0.433
Hospital	0.67 (0.34-1.32)	0.246
Group practice	0.92 (0.44-1.94)	0.829
Other	0.43 (0.15-1.21)	0.109
Multiple settings	0.83 (0.38-1.80)	0.630
**Patients' insurance type**		
Proportion of Medicaid and/or dual coverage patients (<20%)*	Ref.	Ref.
Proportion of Medicaid and/or dual coverage patients (>=20%)	0.85 (0.50-1.45)	0.551
Proportion of uninsured patients (<20%)	Ref.	Ref.
Proportion of uninsured patients (>=20%)	0.95 (0.10-8.86)	0.964

***The variable was omitted due to perfect prediction.

© 2024 Tabata-Kelly M et al. *JAMA Network Open*.

F. Other **(n=903)**

Variables	AOR (95% CI)	P value
**Engagement in HRSN**		
No engagement	Ref.	Ref.
Low-moderate engagement	0.97 (0.47-2.00)	0.936
High engagement	2.21 (1.11-4.37)	0.023
**Age**		
40 and younger	Ref.	Ref.
41-50	1.13 (0.48-2.65)	0.779
51-60	0.85 (0.36-2.03)	0.715
61 and older	0.42 (0.17-1.08)	0.074
**Gender identity**		
Man and Trans man	Ref.	Ref.
Woman and Trans woman	0.72 (0.36-1.45)	0.358
Genderqueer or other	38.61 (5.45-273.39)	p<0.001
**Race/Ethnicity**		
Non-Hispanic White	Ref.	Ref.
Hispanic, Latino or Spanish origin	0.32 (0.03-3.31)	0.342
American Indian or Alaskan Native	0.83 (0.03-24.9)	0.911
Asian	0.73 (0.40-1.35)	0.318
Black or African American	6.94 (1.22-39.62)	0.029
Native Hawaiian or other Pacific Islander	0.02 (0.00-0.47)	0.014
Middle eastern or North African	2.50 (0.86-7.25)	0.091
Multi racial	1.35 (0.33-5.54)	0.681
Other	0.04 (0.01-0.20)	p<0.001
**International Medical Graduates (IMGs)**		
Non-IMG	Ref.	Ref.
IMG	0.61 (0.29-1.29)	0.193
**Engage in teaching and/or research**		
No teaching/research engagement	Ref.	Ref.
Teaching/research engagement	1.09 (0.60-2.00)	0.775
**Geographical locations for providing patient care**		
Rural serving (<20%)	Ref.	Ref.
High rural serving (>=20%)	0.78 (0.37-1.67)	0.522
**Practice settings**		
Private practice	Ref.	Ref.
System	1.57 (0.56-4.40)	0.395
Hospital	0.98 (0.43-2.21)	0.963
Group practice	1.46 (0.60-3.55)	0.403
Other	0.44 (0.14-1.43)	0.174
Multiple settings	0.93 (0.34-2.52)	0.890
**Patients' insurance type**		
Proportion of Medicaid and/or dual coverage patients (<20%)*	Ref.	Ref.

© 2024 Tabata-Kelly M et al. *JAMA Network Open*.

Proportion of Medicaid and/or dual coverage patients (>=20%)	1.00 (0.56-1.82)	0.976
Proportion of uninsured patients (<20%)	Ref.	Ref.
Proportion of uninsured patients (>=20%)	0.78 (0.13-4.73)	0.779

© 2024 Tabata-Kelly M et al. *JAMA Network Open*.

eTable 4: Sensitivity analyses—Multivariate Ordinary Least Squares Regression Analysis of the Association between Physicians' Engagement in Addressing Health-Related Social Needs and Burnout

A. *With Continuous Burnout Outcome*[*] (N=5,358)

Variables	Coefficient (95% CI)	Standard error	P value
**Engagement in HRSN**			
No engagement	Ref.	Ref.	Ref.
Low-moderate engagement	2.45 (-.591, 5.49)	1.55	0.114
High engagement	5.95 (2.23,9.68)	1.9	0.002
**Age**			
40 and younger	Ref.	Ref.	Ref.
41-50	2.17 (-2.67,7.00)	2.47	
51-60	1.47 (-3.20,6.14)	2.38	0.538
61 and older	*-2.81 (-7.47,1.85)	2.38	0.237
**Gender identity**			
Man and Trans man	Ref.	Ref.	Ref.
Woman and Trans woman	2.78 (-0.15,5.71)	1.49	0.063
Genderqueer or other	8.52 (-9.24, 26.29)	9.06	0.347
**Race/Ethnicity**			
Non-Hispanic White	Ref.	Ref.	Ref.
Hispanic, Latino or Spanish origin	*-4.70 (-10.61,1.21)	3.02	0.119
American Indian or Alaskan Native	19.85 (-22.60, 62.30)	21.65	0.359
Asian	*-3.91 (-7.01, -0.81)	1.58	0.013
Black or African American	*-4.03 (-14.11, 6.05)	5.14	0.433
Native Hawaiian or other Pacific Islander	*-16.75 (-23.42, -10.08)	3.40	p<0.001
Middle eastern or North African	5.22 (-3.18, 13.62)	4.29	0.223
Multi racial	4.42 (-11.22, 20.05)	7.97	0.580
Other	*-1.57 (-10.32, 7.17)	4.46	0.724
**International Medical Graduates (IMGs)**			
Non-IMG	Ref.	Ref.	Ref.
IMG	*-3.388 (-7.02, -0.74)	1.61	0.015
**Specialty**			
Medical Specialties	Ref.	Ref.	Ref.
Primary care	2.26 (-1.37, 5.91)	1.86	0.223
Surgery	*-0.37 (-4.37,3.63)	2.04	0.855
Emergency medicine	6.64 (-1.96, 15.25)	4.39	0.130
Psychiatry	*-1.81 (-7.46, 3.84)	2.88	0.530
Other	*-0.25 (-4.47-3.96)	2.15	0.906
**Engage in teaching and/or research**			
No teaching/research engagement	Ref.	Ref.	Ref.
Teaching/research engagement	*-2.48 (-5.48, 0.51)	1.53	0.105
**Geographical locations for providing patient care**			

© 2024 Tabata-Kelly M et al. *JAMA Network Open*.

Variables	Coefficient (95% CI)	Standard error	P value
Rural serving (<20%)	Ref.	Ref.	Ref.
Rural serving (>=20%)	*-1.65 (-5.15, 1.86)	1.79	0.358
**Practice settings**			
Private practice	Ref.	Ref.	Ref.
System	3.62 (-1.17, 8.42)	2.45	0.139
Hospital	1.07 (-3.03, 5.18)	2.09	0.607
Group practice	3.46 (-0.80, 7.71)	2.17	0.112
Other	*-1.49 (-6.19, 3.20)	2.39	0.533
Multiple settings	*-5.22 (-9.09, -1.36)	1.97	0.008
**Patients' insurance type**			
Proportion of Medicaid and/or dual coverage patients (<20%)*	Ref.	Ref.	Ref.
Proportion of Medicaid and/or dual coverage patients (>=20%)	*-1.30 (-3.98, 1.38)	1.37	0.342
Proportion of uninsured patients (<20%)	Ref.	Ref.	Ref.
Proportion of uninsured patients (>=20%)	1.57 (-5.17, 8.30)	3.44	0.648

*The categorical dependent variable, burnout, was converted into continuous variables as follows: Never = 0/365, A few times a year or less = 3/365, Once a month or less = 1/30, A few times a month = 3/30, Once a week = 1/7, A few times a week = 3/7, Everyday = 1. These values were then rescaled to a 0-100% range.
References for this methodology: (1) Robitzsch, Alexander. "Why ordinal variables can (almost) always be treated as continuous variables: Clarifying assumptions of robust continuous and ordinal factor analysis estimation methods." Frontiers in education. Vol. 5. Frontiers Media SA, 2020. (2) Hu, Xiaochu, Lorraine W. Clarke, and Kamran Zendehdel. "Farmers' market usage, fruit and vegetable consumption, meals at home and health–evidence from Washington, DC." Sustainability 13.13 (2021): 7437.

*B. With Ordinal Burnout Outcome** (N=5,358)

Variables	AOR (95% CI)	P value
**Engagement in HRSN**		
No engagement	Ref.	Ref.
Low-moderate engagement	1.30 (1.06-1.58)	0.012
High engagement	1.47 (1.17-1.87)	0.001
**Age**		
40 and younger	Ref.	Ref.
41-50	1.21 (0.92-1.60)	0.178
51-60	0.97 (0.74-1.29)	0.840
61 and older	0.55 (0.42-0.73)	p<0.001
**Gender identity**		
Man and Trans man	Ref.	Ref.
Woman and Trans woman	1.23 (1.03-1.47)	0.024
Genderqueer or other	2.88 (0.83-9.97)	0.094
**Race/Ethnicity**		
Non-Hispanic White	Ref.	Ref.
Hispanic, Latino or Spanish origin	0.75 (0.48-1.15)	0.187

© 2024 Tabata-Kelly M et al. *JAMA Network Open*.

Variables	AOR (95% CI)	P value
American Indian or Alaskan Native	2.75 (0.45-16.69)	0.271
Asian	0.72 (0.59-0.88)	0.001
Black or African American	0.60 (0.31-1.16)	0.128
Native Hawaiian or other Pacific Islander	0.31 (0.14-0.71)	0.006
Middle eastern or North African	1.36 (0.88-2.08)	0.162
Multi racial	1.07 (0.50-2.28)	0.871
Other	0.71 (0.33-1.54)	0.390
**International Medical Graduates (IMGs)**		
Non-IMG	Ref.	Ref.
IMG	0.70 (0.56-0.88)	0.002
**Specialty**		
Medical Specialties	Ref.	Ref.
Primary care	1.18 (0.95-1.47)	0.142
Surgery	0.99 (0.76-1.29)	0.923
Emergency medicine	1.26 (0.80-1.99)	0.310
Psychiatry	0.93 (0.62 - 1.40)	0.726
Other	0.94 (0.72-1.22)	0.642
**Engage in teaching and/or research**		
No teaching/research engagement	Ref.	Ref.
Teaching/research engagement	0.98 (0.89-1.18)	0.807
**Geographical locations for providing patient care**		
Rural serving (<20%)	Ref.	Ref.
Rural serving (>=20%)	0.94 (0.75-1.17)	0.565
**Practice settings**		
Private practice	Ref.	Ref.
System	1.45 (1.10-1.92)	0.009
Hospital	1.29 (0.98-1.70)	0.066
Group practice	1.50 (1.16-1.95)	0.002
Other	1.02 (0.76-1.37)	0.893
Multiple settings	0.92 (0.70-1.21)	0.558
**Patients' insurance type**		
Proportion of Medicaid and/or dual coverage patients (<20%)*	Ref.	Ref.
Proportion of Medicaid and/or dual coverage patients (>=20%)	0.89 (0.75-1.05)	0.175
Proportion of uninsured patients (<20%)	Ref.	Ref.
Proportion of uninsured patients (>=20%)	0.96 (0.61-1.50)	0.852

*Ordinal outcomes of burnout (5-point ordinal scale from never to daily)

© 2024 Tabata-Kelly M et al. *JAMA Network Open*.

C. *With a 5-point ordinal scale of HRSN engagement* **(N=5,358)**

Variables	AOR (95% CI)	P value
**Engagement in HRSN**		
Never	Ref.	Ref.
Less than once a month	1.41 (1.07-1.86)	0.014
Monthly	1.17 (0.81-1.67)	0.405
Weekly	1.72 (1.27-2.34)	0.001
Daily	1.71 (1.21-2.41)	0.002
**Age**		
40 and younger	Ref.	Ref.
41-50	1.15 (0.80-1.65)	0.444
51-60	1.00 (0.70-1.43)	0.990
61 and older	0.59 (0.41-0.86)	0.005
**Gender identity**		
Man and Trans man	Ref.	Ref.
Woman and Trans woman	1.04 (0.84-1.30)	0.699
Genderqueer or other	3.37 (0.75-15.21)	0.114
**Race/Ethnicity**		
Non-Hispanic White	Ref.	Ref.
Hispanic, Latino or Spanish origin	0.50 (0.28-0.89)	0.018
American Indian or Alaskan Native	1.67 (0.15-18.81)	0.677
Asian	0.74 (0.58-0.96)	0.023
Black or African American	0.73 (0.34-1.57)	0.425
Native Hawaiian or other Pacific Islander	0.03 (0.01-0.11)	p<0.001
Middle eastern or North African	1.41 (0.84-2.38)	0.195
Multi racial	0.72 (0.28-1.88)	0.505
Other	0.80 (0.38-1.61)	0.532
**International Medical Graduates (IMGs)**		
Non-IMG	Ref.	Ref.
IMG	0.74 (0.56-0.97)	0.030
**Specialty**		
Medical Specialties	Ref.	Ref.
Primary care	1.22 (0.93-1.63)	0.155
Surgery	1.03 (0.74-1.43)	0.845
Emergency medicine	1.41 (0.84-2.34)	0.190
Psychiatry	1.12 (0.69-1.82)	0.632
Other		
**Engage in teaching and/or research**		
No teaching/research engagement	Ref.	Ref.
Teaching/research engagement	0.81 (0.71-1.16)	0.439
**Geographical locations for providing patient care**		

© 2024 Tabata-Kelly M et al. *JAMA Network Open*.

Variables	AOR (95% CI)	P value
Rural serving (<20%)	Ref.	Ref.
Rural serving (>=20%)	1.07 (0.81-1.40)	0.652
**Practice settings**		
Private practice	Ref.	Ref.
System	1.33 (0.96-1.86)	0.089
Hospital	1.28 (0.93-1.75)	0.131
Group practice	1.47 (1.09-1.98)	0.012
Other	0.96 (0.66-1.40)	0.843
Multiple settings	0.86 (0.59-1.25)	0.426
**Patients' insurance type**		
Proportion of Medicaid and/or dual coverage patients (<20%)*	Ref.	Ref.
Proportion of Medicaid and/or dual coverage patients (>=20%)	0.81 (0.65-1.00)	0.052
Proportion of uninsured patients (<20%)	Ref.	Ref.
Proportion of uninsured patients (>=20%)	1.24 (0.75-2.03)	0.405

D. Inclusion of Physicians Who Responded "Not Certain" for HRSN Engagement **(N=5,804)**

Variables	AOR (95% CI)	P value
**Engagement in HRSN**		
No engagement	Ref.	Ref.
Low-moderate engagement	1.31 (1.02-1.69)	0.034
High engagement	1.70 (1.29-2.23)	<0.001
Not certain	1.09 (0.73-1.62)	0.680
**Age**		
40 and younger	Ref.	Ref.
41-50	1.17 (0.83-1.66)	0.370
51-60	0.96 (0.70-1.39)	0.936
61 and older	0.61 (0.43-0.87)	0.006
**Gender identity**		
Man and Trans man	Ref.	Ref.
Woman and Trans woman		
Genderqueer or other		
**Race/Ethnicity**		
Non-Hispanic White	Ref.	Ref.
Hispanic, Latino or Spanish origin	0.54 (0.31-0.95)	0.034
American Indian or Alaskan Native	1.48 (0.13-17.3)	0.753
Asian	0.78 (0.62-1.00)	0.050
Black or African American	0.67 (0.32-1.39)	0.279
Native Hawaiian or other Pacific Islander	0.03 (0.01-0.11)	<0.001
Middle eastern or North African	1.28 (0.79-2.10)	0.319
Multi racial	1.16 (0.51-2.64)	0.726
Other	0.79 (0.40-1.54)	0.483

© 2024 Tabata-Kelly M et al. *JAMA Network Open*.

**International Medical Graduates (IMGs)**		
Non-IMG	Ref.	Ref.
IMG	0.74 (0.57-0.95)	0.020
**Specialty**		
Medical Specialties	Ref.	Ref.
Primary care	1.20 (0.91-1.57)	0.192
Surgery	0.98 (0.72-1.34)	0.900
Emergency medicine	1.38 (0.84-2.26)	0.204
Psychiatry	1.01 (0.63-1.62)	0.964
Other		
**Engage in teaching and/or research**		
No teaching/research engagement	Ref.	Ref.
Teaching/research engagement	0.92 (0.73-1.16)	0.461
**Geographical locations for providing patient care**		
Rural serving (<20%)	Ref.	Ref.
High rural serving (>=20%)	1.12 (0.85-1.46)	0.418
**Practice settings**		
Private practice	Ref.	Ref.
System	1.46 (1.06-2.00)	0.021
Hospital	1.35 (0.99-1.83)	0.059
Group practice	1.46 (1.09-1.95)	0.011
Other	1.08 (0.75-1.55)	0.679
Multiple settings	0.89 (0.62-1.28)	0.545
**Patients' insurance type**		
Proportion of Medicaid and/or dual coverage patients (<20%)*	Ref.	Ref.
Proportion of Medicaid and/or dual coverage patients (>=20%)	0.79 (0.64-0.98)	0.031
Proportion of uninsured patients (<20%)	Ref.	Ref.
Proportion of uninsured patients (>=20%)	1.15 (0.70-1.90)	0.575

© 2024 Tabata-Kelly M et al. *JAMA Network Open*.
