# Supplementary material for: Physician Engagement in Addressing Health-Related Social Needs and Burnout
Source: JAMA Netw Open. 2024 Dec 30;7(12):e2452152. doi: 10.1001/jamanetworkopen.2024.52152 (PMC11686412; doi:10.1001/jamanetworkopen.2024.52152)
Supplement: Supplement 2. — Data Sharing Statement [file jamanetwopen-e2452152-s002.pdf]

Data Sharing Statement

Tabata-Kelly. Physician Engagement in Addressing Health-Related Social Needs and Burnout. *JAMA Netw Open*. Published December 30, 2024. doi:10.1001/jamanetworkopen.2024.52152

Data
Data available: No
